# Prediction of a New Ligand-Binding Site for Type 2 Motif based on the Crystal Structure of ALG-2 by Dry and Wet Approaches

**DOI:** 10.3390/ijms13067532

**Published:** 2012-06-18

**Authors:** Takeshi Takahashi, Hironori Suzuki, Tatsutoshi Inuzuka, Hideki Shibata, Masatoshi Maki

**Affiliations:** Department of Applied Molecular Biosciences, Graduate School of Bioagricultural Sciences, Nagoya University, Furo-cho, Chikusa-ku, Nagoya 464-8601, Japan; E-Mails: takahashi.takeshi@a.mbox.nagoya-u.ac.jp (T.T.); suzuhiro@post.kek.jp (H.S.); inuzuka.tatsutoshi@f.mbox.nagoya-u.ac.jp (T.I.); shibabou@agr.nagoya-u.ac.jp (H.S.)

**Keywords:** ALG-2, calcium-binding protein, computational prediction, protein-protein interaction, proline-rich motif

## Abstract

ALG-2 is a penta-EF-hand Ca^2+^-binding protein and interacts with a variety of intracellular proteins. Two types of ALG-2-binding motifs have been determined: type 1, P*X*YP*X*nYP (*X*, variable; *n* = 4), in ALIX and PLSCR3; type 2, P*X*PGF, in Sec31A and PLSCR3. The previously solved X-ray crystal structure of the complex between ALG-2 and an ALIX peptide containing type 1 motif showed that the peptide binds to Pocket 1 and Pocket 2. Co-crystallization of ALG-2 and type 2 motif-containing peptides has not been successful. To gain insights into the molecular basis of type 2 motif recognition, we searched for a new hydrophobic cavity by computational algorithms using MetaPocket 2.0 based on 3D structures of ALG-2. The predicted hydrophobic pocket designated Pocket 3 fits with *N*-acetyl-ProAlaProGlyPhe-amide, a virtual penta-peptide derived from one of the two types of ALG-2-binding sites in PLSCR3 (type 2 motif), using the molecular docking software AutoDock Vina. We investigated effects of amino acid substitutions of the predicted binding sites on binding abilities by pulldown assays using glutathione-*S*-transferase -fused ALG-2 of wild-type and mutant proteins and lysates of cells expressing green fluorescent protein -fused PLSCR3 of wild-type and mutants. Substitution of either L52 with Ala or F148 with Ser of ALG-2 caused loss of binding abilities to PLSCR3 lacking type 1 motif but retained those to PLSCR3 lacking type 2 motif, strongly supporting the hypothesis that Pocket 3 is the binding site for type 2 motif.

## 1. Introduction

Eukaryotes have a group of proteins that each possess a domain of five serially repeated helix-loop-helix Ca^2+^-binding motifs (EF-hands). The domain of approximately 170 amino acid residues encompassing the five EF-hand motifs is named a penta-EF-hand (abbreviated PEF) domain [1,2]. In humans, PEF domains are found in more than five proteins: ALG-2, peflin, sorcin, grancalcin and members of the classical calpain family [3]. A unique feature of the PEF is homo-dimerization or hetero-dimerization with closely related PEF proteins. X-ray crystal structural analyses of these PEF proteins revealed that the fifth EF hand (EF5) of each molecule parallels to form a major interface for dimerization [4].

ALG-2 was originally identified as one of the factors associated with cell death of mouse T cell hybridoma induced by stimulants such as T-cell receptor cross-linking, Fas, and glucocorticoid. It was thus named “apoptosis-linked gene 2” [5] and the gene name *PDCD6* has been adopted from “programmed cell death 6”. ALG-2 associates with a number of cellular proteins in a Ca^2+^-dependent manner, such as ALIX [6,7], annexin A7 [8], annexin A11 [9], TSG101 [10], Sec31A [11,12], RBM22 [13], Scotin [14], PLSCR3 [15], PATL1 [16], ASK1 [17], Raf-1 [18] and mucolipin-1 [19]. Except for the last three proteins, ALG-2-interacting proteins have Pro-rich regions. Sequence comparison and mutational analyses of ALG-2-binding sites in ALIX, PLSCR3 and Sec31A have revealed the presence of at least two types of ALG-2-binding motifs in the Pro-rich regions. While type 1 motif (P*X*YP*X*nYP; *X*, variable residue; *n* = 4) is represented in ALIX and PLSCR3, type 2 motif (P*X*PGF) is represented in PLSCR3 and Sec31A [15,20]. Both motifs are found in spatially separated Pro-rich regions (ALG-2-binding site, ABS, 1 and 2) of PLSCR3 and their ALG-2 binding capacities have been experimentally demonstrated [15].

We previously solved the crystal structure of the complex between ALG-2 and a peptide of ALIX containing type 1 motif (ALIX ABS peptide: 799-QGPPYPTYPGYPGYCQ-814; agreement with motif being underlined) in the presence of Zn^2+^ instead of Ca^2+^ [21]. The peptide binds to two juxtaposed hydrophobic pockets named Pocket 1 and Pocket 2, which hold PPYP and YP, respectively. We attempted to co-crystallize ALG-2 and type 2 motif-containing peptides derived from PLSCR3 or Sec31A, but we failed. While the alternatively spliced isoform of ALG-2 (designated ALG-2^ΔGF122^) can bind peptides containing type 2 motif, it does not bind those containing type 1 motif [12,15]. The dissimilarity between the two motifs in the sequence as well as the capacity of binding to two isoforms suggests that type 2 motif is accepted by pockets different from Pocket 1 and Pocket 2. However, locations of new pockets have remained unidentified.

Calmodulin is the most extensively studied EF-hand-type Ca^2+^-binding protein. It shows a gross conformational change upon binding to its target peptides as if the peptides are wrapped around by the N-terminal lobe (EF1–EF2) and C-terminal lobe (EF3–EF4) of calmodulin [22]. On the other hand, the difference between the structure of ALG-2 and that in the complex with the ALIX peptide was found to be small [21]. Assuming that a similar mode of binding is taken by type 2 motif peptides, the Pro-rich motif peptides should bind to cavities or pockets on the protein surface that should be similar with those in the complex. In this study, we predicted a type 2 motif-binding pocket by computational approaches and evaluated the binding site by *in vitro* binding assays using amino acid-substituted mutants of ALG-2.

## 2. Results and Discussion

### 2.1. Prediction of Potential Binding Sites in the Dimer Molecule of ALG-2

**Figure 1:**
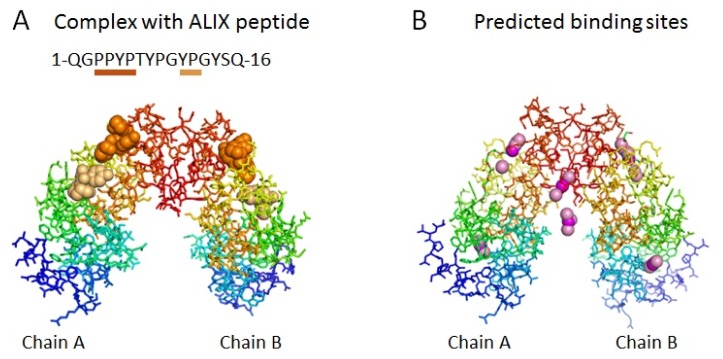
Ligand-binding sites in ALG-2 dimer. (**A**) Crystal structure of the Zn^2+^-bound form of des3-23ALG-2 in the complex with ALIX ABS peptide (PDB ID: 2zne). ALG-2 dimer molecules (chains A and B) are drawn by PyMol and displayed by stick models in rainbow colors (blue to red gradation from *N*-terminal to *C*-terminal regions). Orange and light orange spheres indicate PPYP and YP moieties of the ALIX peptide (underlined) bound to ALG-2. The ALIX peptide bound to chain B is shown for YP from chain D and for PPYP from chain H in two-fold crystallographically symmetric ALG-2 tetramer (chains A, B, E and F) that is in complex with four peptide molecules with two different binding patterns (chains C, D, G and H. see Supplementary file of 2zne_and_symmetry in PDB format). Zinc atoms are not shown. (**B**) Top six ranked ligand-binding sites predicted in the ALG-2 dimer (des3-20ALG-2, PDB ID: 2zn9) by MetaPocket 2.0 using chains A and B of PDB ID 2zn9. Spheres colored in magenta and light pink indicate predicted centers of ligand-binding sites by MetaPocket and top three sites by individual predictors, respectively. Calcium atoms are not shown.

We employed a freely available on-line tool named MetaPocket 2.0 [23], which was designed to predict consensus sites in ranking by combining results of eight individually developed predictors including LIGSITE*^csc^* [24], PASS [25], Q-SiteFinder [26], SURFNET [27], Fpocket [28], GHECOM [29], ConCavity [30] and POCASA [31]. We first examined efficacy of this combined computational approach by comparing the known binding sites in the crystal structure of Zn^2+^-bound form of des3-23ALG-2/ALIX ABS peptide complex (PDB ID: 2zne) with the binding sites predicted by MetaPocket 2.0 using the crystal structure of Ca^2+^-bound form of des3-20ALG-2 (PDB ID: 2zn9). As shown in Figure 1, the ALIX ABS peptides (panel A, orange spheres indicating PPYP and light orange spheres indicating YP, respectively) occupy two of the six predicted binding site clusters in chains A and B of ALG-2 dimer by MetaPocket 2.0 (panel B), demonstrating successful prediction. Two of the additionally predicted sites are formed at a crevice created between each molecule of ALG-2 dimer (chains A and B). Previous mutational analyses of ALG-2 showed that ALG-2^Y180A^ (substitution of Y180 with alanine) lost both the ability to form a homodimer [32] and the ability to bind to ALIX but retained the ability to bind to PLSCR3 and Sec31A [15]. Thus, an authentic binding site for type 2 motif should be unaffected by dimerization. Since the crevice formed between chains A and B of ALG-2 dimer can be excluded, two other predicted sites more proximal to N-terminal regions (cyan to green in the rainbow colors) are promising.

### 2.2. Prediction of Binding Sites for Type 2 Motif in the Monomer Molecule of ALG-2

**Figure 2:**
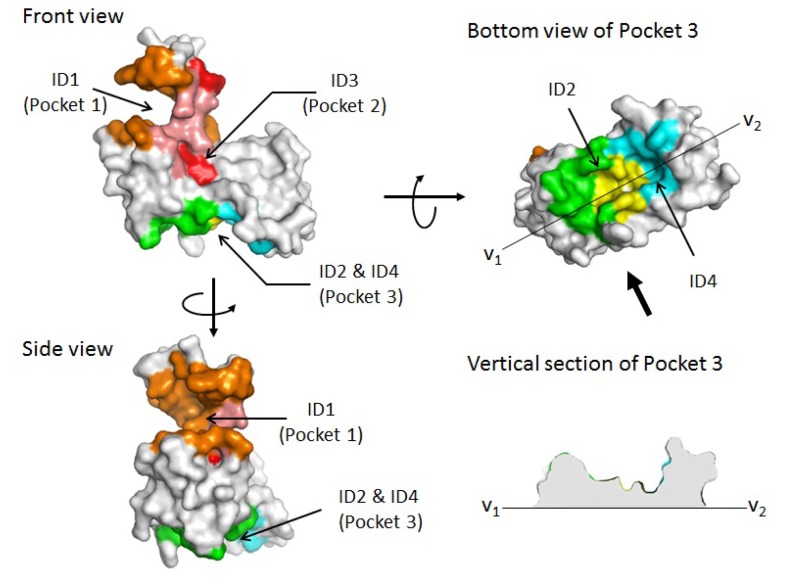
Potential ligand-binding sites predicted by MetaPocket 2.0 in the monomer molecule of ALG-2. Surface of chain A of PDB ID 2zn9 is drawn by PyMol and shown in three different views: front, side and bottom. Residues involved in forming the pockets of predicted potential binding sites (ID Nos. 1–4) are represented by different colors, and pocket numbers are assigned as indicated in Table 2. A view of the vertical section along the line V_1_–V_2_ in Pocket 3 is shown in the right lower panel where interior is painted in light gray.

Prediction of potential binding sites for type 2 motif by MetaPocket 2.0 was further performed by using only chain A of the Ca^2+^-bound form of ALG-2 dimer as a query structure (PDB ID: 2zn9). The top five ranked sites (ID Nos. 1–5) are listed in Table 1. Amino acid residues are partially overlapped between ID Nos. 1 and 3 and between ID Nos. 2 and 4 (Table 1, underlined and double-underlined, respectively), suggesting juxtaposition of these sites. ID Nos. 1 and 3 contain residues known to interact with 3-PPYP and 11-YP of the ALIX peptide (1-QGPPYPTYPGYPGYSQ-16, interacting residues underlined) at previously named Pocket 1 and Pocket 2, respectively, in the crystal structure of the complex (Table 1, letters in magenta) [21]. As shown in the surface presentation in Figure 2, ID No. 1 (Pocket 1) and ID No. 3 (Pocket 2) are juxtaposed (left panels). Binding sites were also predicted in an area distantly located from these sites (bottom of front and side views). Residues of ID No. 2 (green) and ID No. 4 (cyan) partly merge (yellow) and create a pocket named Pocket 3, whose contour displayed at vertical section of line V_1_–V_2_ shows a concave shape (right panels). The relationship between ID numbers of the predicted potential binding sites and pocket numbers designated in this study are summarized in Table 2.

### 2.3. Docking Model of Type 2 Motif Peptide Binding to Hydrophobic Pocket 3

**Figure 3:**
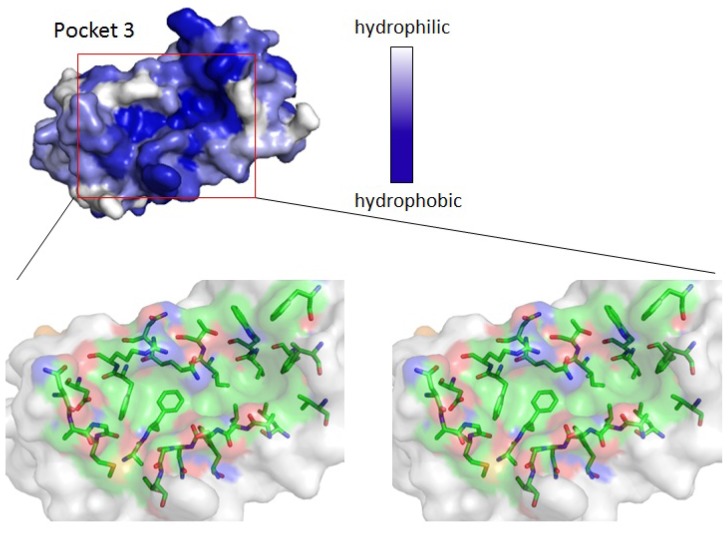
Stereoview of Pocket 3. Surfaces containing the newly designated Pocket 3, corresponding to the predicted binding sites of ID Nos. 2 and 4, are shown and colored according to a hydrophobicity scale (upper panel). Close-up views in stereo (parallel viewing method) are shown and half-transparent surfaces are merged with stick models (bottom panels) of residues involved in the formation of the predicted binding site. Elements are colored only for Pocket 3: carbon, green; oxygen, red; nitrogen, blue; sulfur, orange.

**Figure 4:**
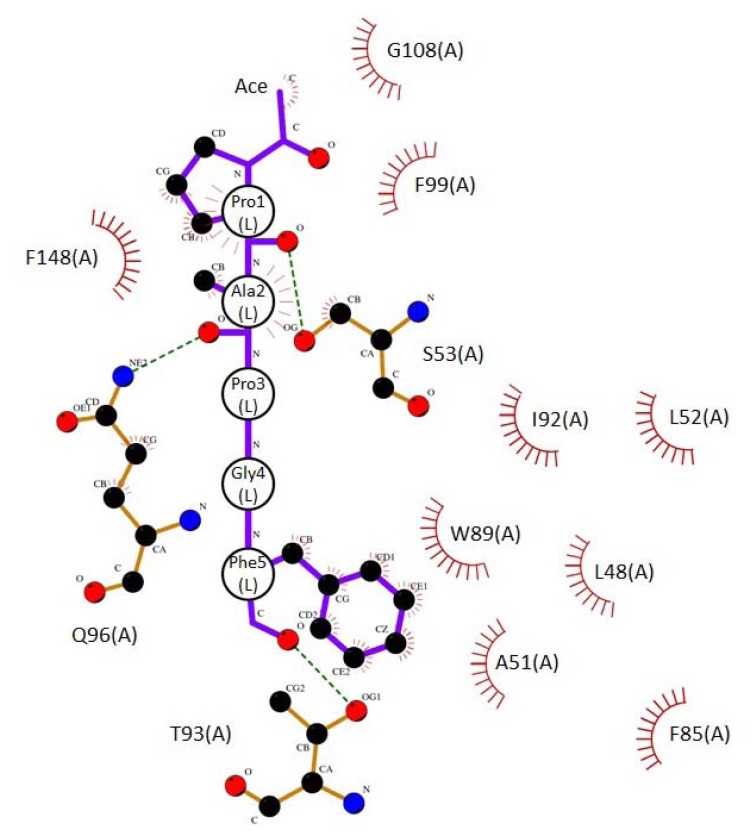
Schematic diagram of interactions between ALG-2 and the model peptide generated by LIGPLOT. *N*-acetyl-ProAlaProGlyPhe-amide, a virtual type 2 motif penta-peptide ligand derived from PLSCR3, was subjected to docking simulation for binding to Pocket 3 by AutoDock Vina, and residues with interactions between a ligand and ALG-2 are schematically displayed by LIGPLOT. Amino acid residues are represented by one and three letter codes for ALG-2 (A) and ligand (L), respectively. Atoms of carbon, oxygen and nitrogen are indicated by small closed circles in black, red and blue, respectively. Hydrogen bonds are indicated by green broken lines. Hydrophobic contacts are indicated by quarter open circles (ALG-2) and small black closed circles (carbon atoms of ligand) with reddish prickles. Ace, acetyl group.

**Figure 5:**
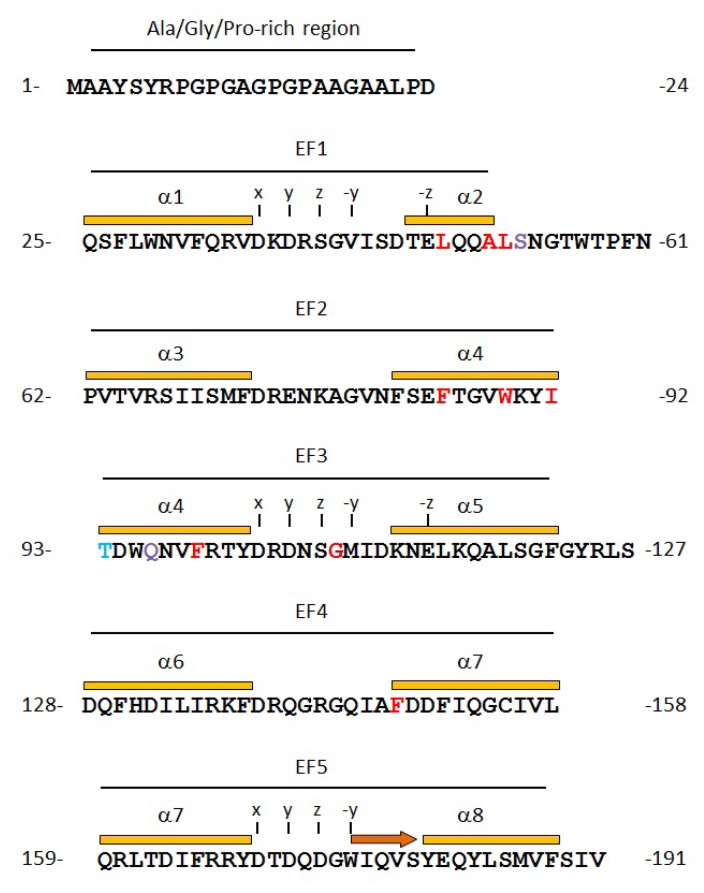
Distributions of the predicted ligand-interacting residues in the primary structure of ALG-2. ALG-2 amino acid residues predicted to interact with the virtual peptide *N*-acetyl-ProAlaProGlyPhe-amide are colored in red, cyan and purple for hydrophobic interactions, hydrogen bonds and both, respectively. Each helix-loop-helix structural motif of EF-hands is marked from EF1 to EF5. Eight α-helices (α1 to α8) and one β-sheet are indicated by orange bars and a dark orange arrow above the sequences. Calcium ions are coordinated with oxygen atoms at positions *x*, *y*, *z*, −*y* and −*z* from amino acid residues and with an oxygen atom at position −*x* from the neighboring water molecule.

**Figure 6:**
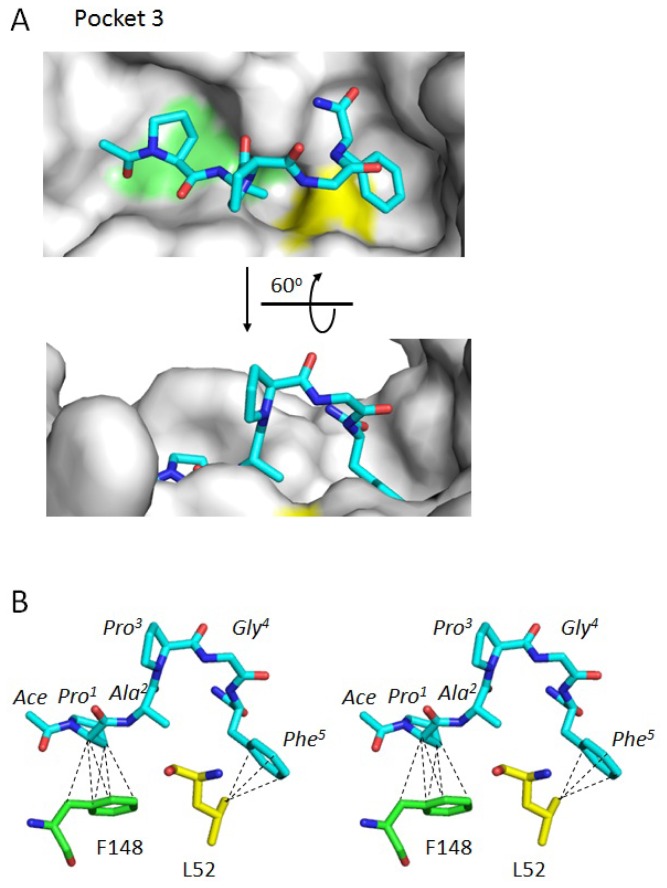
Docking simulation. (**A**) Residues forming Pocket 3 and its vicinity are shown by surface representation in gray except for F148 in light green and L52 in yellow. The type 2 motif peptide is represented by a stick model in which atoms of carbon, nitrogen and oxygen are shown in cyan, blue and red, respectively, in the upper panel. A side view of the structure shown in the upper panel obtained by rotating 60° around the indicated axis is presented in the lower panel; (**B**) The peptide shown in (**A**) upper panel is rotated 75° around the axis and represented in stereoview (parallel viewing method). Hydrophobic interactions between the type 2 motif peptide and ALG-2 are shown for F148 (carbon atoms, green) and L52 (carbon atoms, yellow). Broken lines indicate hydrophobic interactions with distances shorter than or equal to 3.9 Å (see Table 3 for details).

Side chains of amino acid residues comprising type 2 motif and those of neighboring residues are hydrophobic in PLSCR3 (41-QVPAPAPGFALFPS-54, ABM-2 underlined) and Sec31A (837-NPPPPGFIMH-846), suggesting hydrophobic interactions for binding between these ligands and ALG-2. As shown in the upper panel Figure 3, Pocket 3 has a high degree of hydrophobicity and it is created by amino acid residues with hydrophobic side chains (lower panels shown in stereoview). We constructed a ligand binding model in which Pocket 3 accepts *N*-acetyl-Pro^1^Ala^2^Pro^3^Gly^4^Phe^5^-amide, a virtual penta-peptide of a type 2 motif sequence derived from PLSCR3, by using the docking simulation program AutoDock Vina [33]. The calculated lowest energy model (−7.8 kcal/mol; Supplementary file of DockModel_Scr3_ABM-2 in PDB format) was analyzed for protein-ligand interactions by LIGPLOT [34] and NCONT [35]. A schematic diagram is shown in Figure 4, and interactions are listed in Table 3. In this docking model, carbon atoms of Pro^1^, Ala^2^ and Phe^5^ of the ligand interact with side chains of L48, A51, L52, S53, F85, W89, I92, Q96 and F148. The carbon atom from the methyl moiety of the acetyl group also interacts hydrophobically with F99 and G108. On the other hand, hydrogen bonds are predicted to be formed between oxygen atoms of peptide bonds (Pro^1^, Ala^2^ and Phe^5^) and oxygen atoms and a nitrogen atom of Pocket 3 residues in ALG-2 (S53, T93 and Q96). Moreover, a nitrogen atom of the *C*-terminal amide forms a hydrogen bond with an oxygen atom in the side chain of Gln96 (Table 3, not shown in Figure 4). As shown in Figure 5, interacting residues are from three segments in the ALG-2 primary structure: (i) α-helix 2 (exiting helix of EF1) and the following loop (L48, A51, L52, S53); (ii) α-helix 4 that is continuous from the exiting helix of EF2 and entering helix of EF3 (F85, W89, I92, T93, Q96 and F99) and EF3 loop, and (iii) the start of α-helix 7 (F148). As shown in Figure 6, the model peptide fits well into Pocket 3. While Pro^3^ and Phe^5^ of the peptide settle to the bottom by interacting with L52 (colored in yellow) and F148 (colored in light green), the linker segment, Pro^3^Gly^4^, loops out from the cavity.

The crystal structure of ALG-2 in the Ca^2+^-bound form (PDB ID 2zn9) shows that Pocket 3 is largely covered with a crystallographic symmetry-related monomer (not the partner of dimer, Supplementary Figure S1). Inaccessibility of the ABS-2 peptide to Pocket 3 in the crystal might explain why we could not obtain the crystal structure of the complex between ALG-2 and the ABS-2 peptide. Masking of Pocket 3 by another ALG-2 molecule, however, may happen only in the crystal and in solution, if it happens at all, at a very high concentration of ALG-2. Pocket 3 should be opened to the ligand in the cell and in solution under experimentally controlled conditions.

### 2.4. *In Vitro* Binding Assay

**Figure 7:**
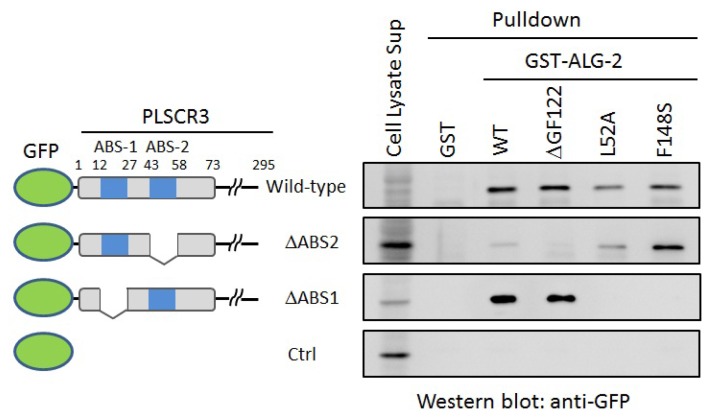
Effects of amino acid substitutions in Pocket 3 on the binding abilities to PLSCR3 by GST-ALG-2 pulldown assays. HEK293/ALG-2_KD_ cells were transfected with expression vectors for GFP-fused PLSCR3 of wild-type, ABS2-deletion mutant (ΔABS2), ABS1-deletion mutant (ΔABS1) and unfused GFP used as a negative control (Ctrl). Supernatants of cell lysates were subjected to pulldown assays using GST-fused ALG-2 proteins of wild-type (WT), alternatively spliced isoform lacking Gly^121^Phe^122^ (ΔGF122), mutant of Leu^52^ to alanine substitution (L52A) and mutant of Phe^148^ to serine substitution (F148S) as well as unfused GST used as a negative control in the presence of 100 μM CaCl_2_. Proteins bound to the glutathione Sepharose beads (pulldown fractions) were analyzed by Western blotting using an anti-GFP antibody. Protein samples corresponding to 1% of total cleared cell lysates (Cell Lysate Sup) and 20% of pulldown fractions (Pulldown) were loaded to each lane on 10% gels of SDS-PAGE.

To investigate the possibility that Pocket 3 is a genuine binding site for type 2 motif as predicted, we performed *in vitro* binding assays using amino acid-substituted mutants of ALG-2. Since a high concentration of ALG-2 (~5 μM) tends to aggregate in the presence of Ca^2+^ [36] and hampers analyses of protein-ligand interaction. Addition of non-ionic detergent alleviates the problem of aggregation but limits the methods to be employed for interaction analyses. Here we employed the glutathione-*S-*transferase (GST) pulldown method. Immobilization of the GST-fused ALG-2 proteins on glutathione Sepharose beads was thought to be effective to avoid the potential masking problem of Pocket 3 by neighboring ALG-2 molecule as described above. Among the predicted interacting residues of ALG-2, L52 and F148 were targeted for amino acid substitutions (L52, alanine; F148, serine) since they are located in the boundary of helices and loops and adverse effects on the secondary structures were presumed to be minimal (Figure 5). Supernatants of the lysates of cells expressing green fluorescent protein (GFP)-fused PLSCR3 of wild-type and mutants lacking either ABS-1 or ABS-2 were subjected to GST-pulldown assays in the presence of 100 μM CaCl^2^ and 0.1% Triton X-100, and proteins bound to the glutathione Sepharose beads were analyzed by Western blotting using an anti-GFP antibody. As shown in Figure 7, while GFP-Scr3^ΔABS1^ (retaining type 2 motif) was not detected in the pulldown fractions of GST-ALG-2^L52A^ and GST-ALG-2^F148S^, GFP-Scr3^ΔABS2^ (retaining type 1 motif) was detected in the case of these ALG-2 mutants. Retaining of the binding ability to type 1 motif suggests that structural adverse effects caused by amino acid substitutions of L52 and F148 are locally limited to Pocket 3 and, if any, its surroundings. In contrast, GST-ALG-2^ΔGF122^ showed opposite results: binding to GFP-Scr3^ΔABS1^ but not to GFP-Scr3^ΔABS2^. Unexpectedly, wild-type GST-ALG-2 showed a poorer binding ability to GFP-Scr3^ΔABS2^ than mutant ALG-2 proteins (2nd panel from the top). ABS-1 may have a weaker binding ability than ABS-2, but mutations of Pocket 3 might somehow favored ALG-2 with a stronger binding to ABS1, possibly by avoiding competition with endogenous type 2 motif-containing proteins including Sec31A. In other words, occupation of Pocket 3 might inhibit binding of type 1 motif-containing proteins to Pocket 1 and 2 in the same monomer molecule probably due to steric hindrance. No significant signals for GFP-Scr3 were detected in pulldown fractions of the wild-type and mutant GST-ALG-2 proteins in the presence of 5 mM EGTA, a Ca^2+^ chelator, (Supplementary Figure S2), indicating that mutations of L52 and F148 did not abrogate the Ca^2+^-dependency of interaction. In the second and third lowest energy models (−6.8 kcal/mol and −6.5 kcal/mol, respectively) obtained by the ligand binding simulation with AutoDock Vina, the peptide binds to a part of Pocket 2, and neither L52 nor F148 interacts with the model peptides (data not shown). Thus, the results strongly suggest that Pocket 3 is the most probable candidate for the type 2 motif-binding site and that the two types of ALG-2-binding motifs are accepted by different pockets in ALG-2.

### 2.5. Ca^2+^-Dependent Interaction

Previous studies on crystal structures of the Ca^2+^-bound form of ALG-2 revealed binding of Ca^2+^ to EF1, EF3 and EF5 [21,37], among which the structure of EF1 was found not to be changed by Ca^2+^ binding in a comparison of the 3D structures with the metal-free form [21]. On the other hand, comparison of the metal-free form and the Ca^2+^-bound form of ALG-2 revealed a difference in configuration of the side chain of R125 that is present in the loop connecting EF3 and EF4 and interacts with the ALIX peptide in Pocket 1. Since EF5 has an incomplete Ca^2+^-coordination at the −*z* position (water molecule oxygen atom instead of bidentate side chain oxygen atoms of glutamic acid), its binding affinity seems to be lower than affinities of the other two EF-hands [21]. Assuming that binding of Ca^2+^ to EF3 is the most critical step for ALG-2 to accept ligands, we proposed a Ca^2+^/EF3-induced R125 switch model to explain Ca^2+^-dependent interaction between ALG-2 and ALIX [21]. This model, however, is not applicable to explain the Ca^2+^-dependent binding of type 2 motif peptides, and it remains to be clarified how the signal of binding of Ca^2+^ to EF3 is transduced to Pocket 3. Interestingly, some residues predicted to interact with the virtual type 2 motif peptide are localized in α-helix 4 that forms a part of EF2 and EF3 (Table 3, Figure 5), suggesting that EF3 is somehow involved in the Ca^2+^-dependency for interaction with type 2 motif-containing proteins. Although attempts of co-crystallization of ALG-2 in the complex with the type 2 motif peptides of PLSCR3 and Sec31A have failed, the new finding of Pocket 3 as the most probable candidate of the type 2 motif-binding site provides us a new idea for designing a ligand and/or mutant ALG-2 to facilitate crystallization of the complex for X-ray crystallography, and a definite solution to the Ca^2+^-dependent type 2 motif-binding mechanism will be obtained in the future.

## 3. Experimental Section

### 3.1. Prediction of Potential Binding Sites and Docking Simulation

A program of binding site predictor MetaPocket 2.0 [23] was run online [38]. A molecular visualization tool, PyMol [39], was used for structure representations and measurement of distances between atoms. For surface representation of hydrophobicity by PyMol, rTools 0.7.2 [40] was down-loaded and protscale was selected from Plugin. A 3D-structural model of the virtual peptide of *N*-acetyl-ProAlaProGlyPhe-amide containing type 2 motif was first constructed with COOT [41] and used for ligand docking simulation program AutoDock Vina [33,42]. The program was run by fixing the grids so that they covered entire Pocket 3 and also a part of Pocket 2, which is located at the back side of Pocket 3. Protein-ligand interactions were analyzed using the program LIGPLOT v.4.5.3 [34,43], and selected by NCONT in the CCP4 suite [35].

### 3.2. Expression and Purification of Mutant ALG-2 Proteins

Mutations of nucleotides for amino acid substitutions in ALG-2 were performed by the PCR-based mutagenesis method using pairs of specific primers (L52A substitution: forward: 5′-GCTTCAGCAAGCTGCCTCCAACGGCACGTG-3′; reverse: 5′-CACGTGCCGTTGGAGGCAGC TTGCTGAAGC-3′; F148S substitution: forward: 5′-GGGCAGATCGCCAGCGACGACTTCATC CAG-3′; reverse: 5′-TGGATGAAGTCGTCGCTGGCGATCTGCCC-3′), an expression plasmid of GST-fused human ALG-2 (pGEX-4T-3/hALG-2) [12] as a template and KOD plus DNA polymerase (Toyobo, Osaka, Japan) essentially according to the instruction manual of a QuikChange Site Directed Mutagenesis Kit (Agilent Technologies, Santa Clara CA 95051, USA). After being digested with *Dpn*I (Takara Bio Inc, Osaka, Japan), PCR products were used for transformation of *Escherichia coli* Top10. Isolated plasmid DNAs were subjected to DNA sequencing. After confirming nucleotide substitutions at the targeted sites but no changes in the rest of the ALG-2 cDNA sequence, *Escherichia coli* BL21 cells were transformed with the plasmids, and mutant ALG-2 proteins were expressed and affinity-purified using glutathione Sepharose beads as described elsewhere. Eluted ALG-2 proteins were dialyzed against 20 mM Tris-HCl, pH 7.5, 10 μM EDTA, 10 μM EGTA, and 100 mM NaCl and re-adsorbed to Sepharose beads.

### 3.3. GST-Pulldown Assays

An ALG-2 knocked down cell line (designated HEK293/ALG-2_KD_) [32] that was established previously by the RNA interference method was used to minimize effects of competition on target binding between endogenous ALG-2 present in the cells and GST-ALG-2 immobilized on Sepharose beads. HEK293/ALG-2_KD_ cells were transfected with expression plasmids for GFP-fusion proteins of wild-type and deletion mutants of PLSCR3 by the conventional calcium phosphate precipitation method. One day after transfection, harvested cells were lysed with buffer A (50 mM Tris-HCl, pH 7.5, 150 mM NaCl, 1.5 mM MgCl_2_, 0.1% Triton X-100 and protease inhibitors), and supernatants obtained after centrifugation at 14,000 *g* for 10 min at 4 °C were subjected to GST-pulldown assays essentially as described previously after addition of CaCl_2_ to 100 μM [10,12]. Briefly, Sepharose beads immobilizing GST-ALG-2 proteins were incubated with cleared cell lysates at 4 °C overnight. After Sepharose beads had been recovered by low-speed centrifugation (700 *g*) for 1 min and washed three times with buffer A containing 100 μM CaCl_2_, proteins bound to the beads were subjected to SDS-PAGE followed by Western blot analysis. Proteins transferred to polyvinylidene difluoride (PVDF) membranes (Immobilon-P, Millipore, Bedford, MA, USA) were probed with an anti-GFP monoclonal antibody (clone B-2, Santa Cruz Biotechnology, Santa Cruz, CA, USA). Signals of Western blotting were detected by the chemiluminescence method using Super Signal West Pico Chemiluminescent Substrate (Thermo Fisher Scientific Inc., IL, USA) and analyzed with LAS-3000mini (Fujifilm, Tokyo, Japan).

## 4. Conclusions

In conclusion, a type 2 motif-binding site in ALG-2 was predicted by using a binding-site prediction program. Docking simulation enabled creation of amino acid substitution mutants that were examined by *in vitro* binding experiments. These mutants had lost type 2 motif-binding ability but retained type 1 motif-binding ability, suggesting the presence of motif-specific ligand-binding sites.

## Figures and Tables

**Table 1:**
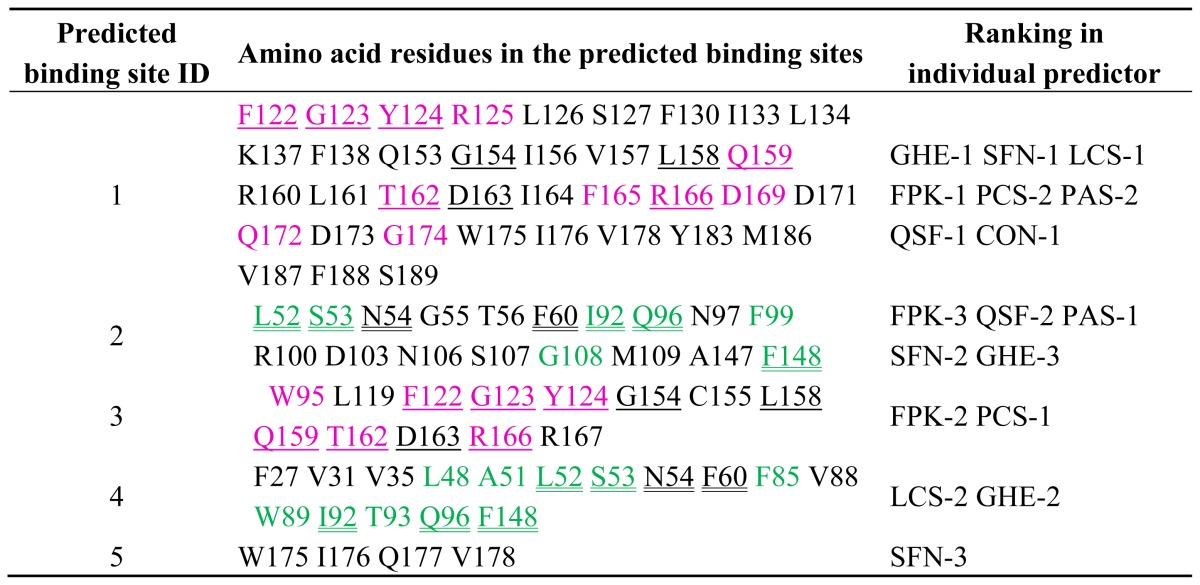
Potential ligand-binding sites in the monomer molecule of ALG-2 predicted by multiple computational algorithms. Chain A of PDB ID 2zn9 was searched for potential ligand-binding sites using MetaPocket 2.0 that combines the results of eight predicting methods. Predicted top five ranked binding sites (ID Nos. 1–5) and the residue numbers of amino acids that form the potential binding sites are listed. Letters in magenta in binding site ID Nos. 1 and 3 are residues known to interact with the ALIX peptide in the crystal structure (PDB ID 2zne, chains A and C). Letters in green in binding sites ID Nos. 2 and 4 are residues predicted to interact with a model type 2 motif peptide by simulation with AutoDock Vina and analyzed with LIGPLOT (See Table 3). Each residue overlapping between ID Nos. 1 and 3 and between ID Nos. 2 and 4 is indicated by an underline and double underline, respectively. Number following the hyphen indicates ranking in individual predictors used in MetaPocket 2.0: ConCavity (CON); Fpocket (FPK); GHECOM (GHE); LigsiteCS (LCS); PASS11 (PAS); POCASA (PCS); Q_SiteFinder (QSF); SURFNET (SFN).

**Table 2:** Relationship between predicted binding site ID numbers and pocket numbers assigned in this study.

Color		Predicted binding site ID number	Pocket number
orange		1	Pocket 1
red		3	Pocket 2
salmon		1 & 3	Pockets 1 & 2
green		2	Pocket 3
cyan		4	Pocket 3
yellow		2 & 4	Pocket 3

**Table 3:** Hydrophobic interactions and hydrogen bonds between a virtual *N*-acetyl-ProAlaProGlyPhe-amide peptide and ALG-2.

Interacting atoms in peptide		Interacting atoms in ALG-2	Distance (Å)
Hydrophobic			
ACE	C a	CE2^F99^	3.7
		CA^G108^	3.6
Pro^1^	CG	CB^Q96^	3.9
	CB	CG^Q96^	3.7
	CG	CG^Q96^	3.9
	CA	CD2^F148^	3.9
	CB	CD2^F148^	3.5
	CB	CE2^F148^	3.8
	CB	CG^F148^	3.7
Ala^2^	CA	CB^S53^	3.5
	CB	CB^S53^	3.5
Phe^5^	CZ	CD1^L48^	3.8
	CE2	CB^A51^	3.6
	CD2	CD2^L52^	3.8
	CE2	CD2^L52^	3.7
	CZ	CD2^L52^	3.9
	CE1	CE1^F85^	3.8
	CZ	CE1^F85^	3.5
	CZ	CZ^F85^	3.7
	CD1	CD1^W89^	3.8
	CE1	CA^W89^	3.8
	CB	CG2^I92^	3.7
	CD1	CG2^I92^	3.6
	CG	CG2^I92^	3.8
Hydrogen bonds			
Pro^1^	O	OG^S53^	3.1
Ala^2^	O	NE2^Q96^	3.1
Phe^5^	O	OG1^T93^	3.0
NH^2^	N	OE1^Q96^	3.0

a Carbon atom from methyl moiety of acetyl group.
